# Limited efficacy of pixantrone in refractory diffuse large B-cell lymphoma

**DOI:** 10.3892/ol.2020.11288

**Published:** 2020-01-10

**Authors:** Aleksander Novakovic, Lucka Boltezar, Barbara Jezersek Novakovic

**Affiliations:** 1 Medical Faculty, University of Ljubljana, 1000 Ljubljana, Slovenia; 2 Department of Lymphoma Treatment, Division of Medical Oncology, Institute of Oncology Ljubljana, 1000 Ljubljana, Slovenia

**Keywords:** relapsed and refractory diffuse large B cell lymphoma, overall survival, pixantrone treatment, effectiveness, anthracycline resistance

## Abstract

Relapsed or refractory diffuse large B-cell lymphoma (DLBCL) is an aggressive disease with poor outcomes in patients ineligible for autologous stem cell transplantation. In this setting, novel treatment approaches are urgently required and the innovative agent pixantrone has shown some promising results in terms of disease-free and overall survival (OS). The present study retrospectively analyzed 12 patients routinely treated with pixantrone in monotherapy or in combinations at the Institute of Oncology Ljubljana, Slovenia, between January 2016 and October 2018. All 12 patients had refractory lymphoma to last treatment and a large proportion of them had other high risk features (high proliferation index, high disease stage, high international prognostic index (IPI) score, high percentage of primary refractory disease and high percentage of refractoriness to anthracyclines) at initiation of pixantrone. All patients progressed during treatment and none of the patients were alive at the time of analysis due to progressive lymphoma. Pixantrone specific median OS was 3.5 months (range, 0.5–10 months). A somewhat superior median OS (P=0.065) was observed in patients primarily sensitive to anthracyclines. Pixantrone has shown only limited efficacy in the present real world study comparable to the results of another real world UK retrospective analysis and substantially worse than the efficacy observed in the PIX301 registration trial. Therefore, an appropriate selection of patients for this treatment is crucial. Despite the limited experience due to a small number of patients, it was recommended to consider only patients with relapsed (and not refractory) disease, patients with non-primary refractory disease and those with fewer lines of prior therapy.

## Introduction

The diffuse large B-cell lymphoma (DLBCL) is the most common subtype of non-Hodgkin's lymphoma (NHL). Its first line treatment usually consists of an anthracycline and rituximab containing regimen while second line salvage therapy in relapsing and refractory patients habitually comprises a platinum and/or gemcitabine based regimen followed by consolidative high dose therapy and autologous stem cell transplantation in younger, fit patients. However, there is no consensus regarding the third and further line therapies in multiply relapsed or refractory aggressive B-cell NHL which typically consist of polychemotherapy combinations excluding the anthracyclines or monotherapy with oxaliplatin, etoposide, gemcitabine and vinorelbine (1–4).

Pixantrone is a novel aza-anthracenedione developed to reduce the risk of cardiotoxicity while maintaining efficacy in the treatment of aggressive lymphomas (5–8). It targets DNA topoisomerase IIα and produces semiquinone free radicals in an enzymatic reducing system. Pixantrone is 10- to 12-fold less damaging to neonatal rat myocytes than doxorubicin or mitoxantrone, as measured by lactate dehydrogenase release. Three factors potentially contribute to the reduced cardiotoxicity of pixantrone-its lack of binding to iron (III) makes it unable to induce iron-based oxidative stress, its low cellular uptake limits its ability to produce semiquinone free radicals and cause a futile redox cycle, and its selectivity for topoisomerase IIα over topoisomerase IIβ (which predominates in postmitotic cardiomyocytes) (9,10).

In a phase II study including patients with relapsed aggressive NHL, an overall response rate of 27% has been shown with pixantrone in monotherapy demonstrating the longest response duration of 24 months (9). A randomized, open-label, multinational phase III study called PIX301 disclosed that pixantrone monotherapy was superior to other monotherapies, according to physician's choice in patients with multiply relapsed or refractory aggressive NHL in terms of overall response rate, median progression free as well as overall survival (OS) (11). The median OS was reported to be 2.6 months longer in the pixantrone arm (11). A recently published extended survival analysis reported that some of the patients achieving a complete or unconfirmed complete response, at the end of the PIX301 trial, survived >400 days without progression (12).

However, the real world post-approval data on the efficacy of pixantrone are, firstly, more than scarce and, secondly, less promising for non-select every day relapsed and refractory patients (13) than the ones reported in the pivotal PIX301 study (11). In fact, it appears that the real world multiply refractory and otherwise high risk patients might have probably been underrepresented in the PIX301 analysis (11). The aim of our retrospective real world analysis was therefore to correlate our experience with pixantrone to the results of the PIX301 trial (11) and to the UK multicenter retrospective real world analysis (13) with an emphasis on patient's characteristics.

## Patients and methods

### 

#### Patients, data collection and treatment

We retrospectively analyzed all the patients routinely treated with pixantrone at the Institute of Oncology Ljubljana, Slovenia. Pixantrone is being administered in Slovenia since January 2016. Our analysis included patients receiving monotherapy with pixantrone as well as those treated with a pixantrone based multidrug regimen [PREBEN-pixantrone, rituximab, bendamustin and etoposide; or PEBEN-pixantrone, etoposide, bendamustin (14)]. Data regarding their lymphoma type, age, stage, international prognostic index (IPI), prior therapy lines and survival were collected from the patients' records. The OS was defined as the time from the first lymphoma diagnosis to the documented death by any cause or the end of observation. The pixantrone specific survival was defined as the time between the first administration of pixantrone and the death by any cause or the end of observation.

Patients were either treated with pixantrone in monotherapy 50 mg/m^2^ on days 1, 8 and 15 or with pixantrone 50 mg/m^2^ on days 1 and 8, combined with rituximab 375 mg/m^2^ on day 1, etoposide 100 mg/m^2^ on day 1, bendamustin 90 mg/m^2^ on day 1 and methylprednisolone, according to the PREBEN protocol (14). In case the relapse was CD20-negative, rituximab was omitted. All patients received granulocyte colony-stimulating factors in pegylated form.

#### Histopathological evaluation

With the intention to further investigate the histopathological characteristics of patients' lymph node samples, we classified the samples according to the Hans algorithm into germinal center B-cell (GCB) and activated B-cell (ABC) subtypes and re-determined CD20 and BCL2 expression as well as the proliferative activity defined with Ki67 expression.

#### Ethical considerations

All procedures followed in this study were in accordance with the ethical standards of the responsible committee on human experimentation (institutional and national) and the Helsinki Declaration of 1975, as revised in 2000. Individual patient consent was not collected for this study as this was a retrospective database analysis and the institutional informed consent form for treatment included consent to use the patient's data, materials and/or test results for research purposes. The study was approved as such as a part of treatment result evaluation (ERID-KESOPKR/17; OIRIKE00038) by the institutional review board of the Institute of Oncology Ljubljana.

#### Statistical analysis

Survival data were analyzed using the Kaplan-Meier survival curves and log-rank test was applied to compare the survival distributions between groups. P<0.05 was considered to indicate a statistically significant difference. The GraphPad Prism program (version 3.02, GraphPad Software) was used for statistical analysis.

## Results

### 

#### Patient characteristics and histopathological data

Three male patients and 9 female patients treated with pixantrone were identified in the database. Their median age at first diagnosis was 65 years, range 36–77 years (Table I). According to the pathology reports, one patient was diagnosed with an Asian variant of intravascular large B-cell lymphoma, 10 patients with the diffuse large B-cell lymphoma not otherwise specified (DLBCL NOS) (6 of them having a variant with high proliferation index) and 1 patient with low grade follicular lymphoma, which later in the disease course transformed into DLBCL. Five (42%) of the patients were determined to have the ABC subtype of DLBCL according to Hans algorithm, all of them being CD20 as well as BCL2-positive and with proliferative activity of 70 to 100%. One of these samples was marked as a double expressor DLBCL. The GCB subtypes were again all positive for CD20 (except one, which was just marginally positive-simultaneously one lymph node sample was CD20-negative, while the other lymph node sample was positive disproportionately in <50% of cells) and for BCL2, while having proliferative activity of 40 to >95%. None of them was marked as double or triple hit lymphoma, but *c-myc* rearrangement was found in 2 samples (Table I).

Eleven patients had stage IV of the disease at presentation and the patient with follicular lymphoma had stage III with involvement of the spleen (stage IIIS). As much as 75% of the patients presented with constitutional symptoms. The IPI score at lymphoma diagnosis was ≥2 in all 12 patients and ≥3 prior to pixantrone treatment.

#### Previous treatment

Eight patients were initially treated with R-CHOP (rituximab, cyclophosphamide, doxorubicin, vincristine, prednisolone), 1 patient with R-CHOEP (rituximab, cyclophosphamide, doxorubicin, vincristine, etoposide, prednisolone), the patient with follicular lymphoma received only CHOP in 1999, 1 patient was treated with a combination of R-CHOP and MD MTX (middle dose methotrexate) and 1 patient received one cycle of R-CHOP with MD MTX and continued her first treatment with R-EPOCH (rituximab, etoposide, prednisolone, vincristine, cyclophosphamide, doxorubicin) (Table I). The patient with primary follicular lymphoma had a long lasting remission following the first CHOP treatment in 1999 and was included in this study after the subsequent transformation into diffuse large B cell lymphoma at the end of 2015.

With aforementioned first line treatments 7 patients achieved complete remission (58%), 2 patients partial remission (17%) and 3 patients progressed during the first line treatment (25%). The median duration of response to first line treatment was 4 months (range, 2–29 months). Regarding the duration of response to first line anthracycline containing regimen, altogether 7 patients (58%) had primary refractory disease. Only 3 patients fulfilled the criteria for primary anthracycline sensitivity according to the PIX301 study.

The prognostic features of our group of patients, which was also the smallest, were the worst compared both to the UK retrospective analysis and especially to the PIX301 study cohort as given in Table II. All patients received rituximab prior to pixantrone treatment-11 in first line treatment and 1 patient in second, fourth, fifth and seventh line of treatment. In two patients, the relapse at the time of pixantrone treatment was confirmed to be CD20-negative. All 12 patients were also classified to have refractory disease to last treatment prior to pixantrone according to PIX301 criteria.

#### Pixantrone treatment

**Figure 1.**
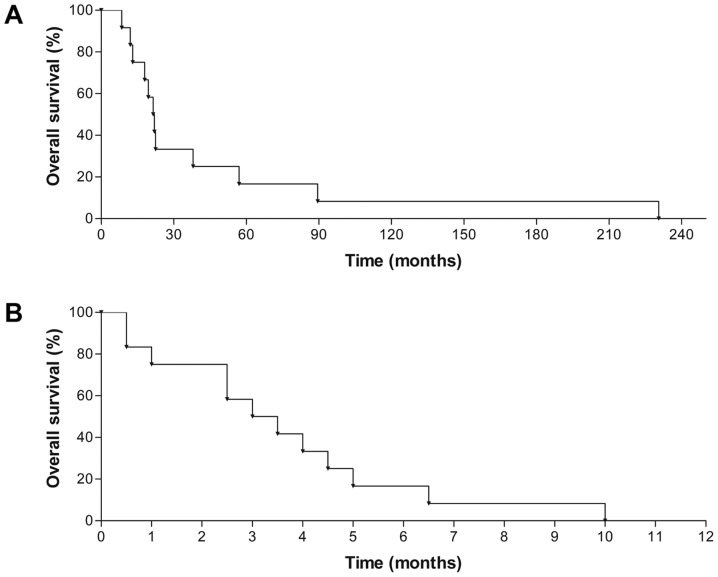
Median OS of pixantrone-treated patients. (A) Median OS from lymphoma diagnosis. (B) Pixantrone-specific median OS. OS, overall survival.

**Figure 2.**
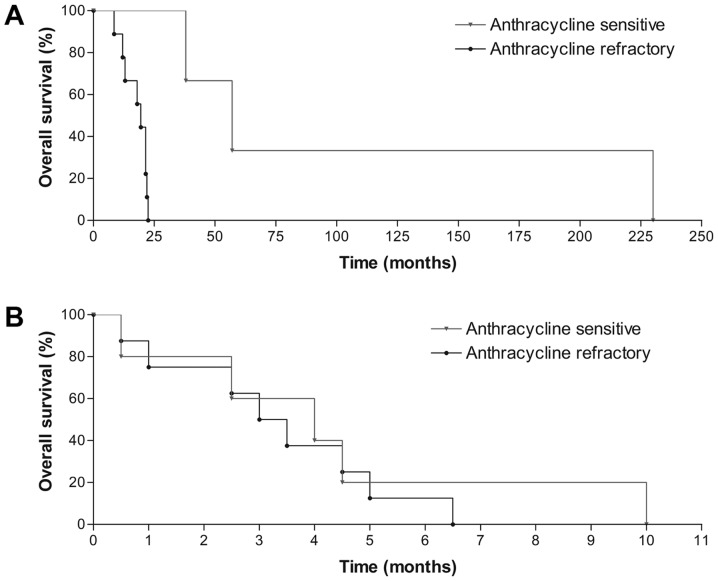
Median OS of pixantrone-treated patients according to anthracycline sensitivity. (A) Median OS from lymphoma diagnosis according to anthracycline sensitivity. (B) Pixantrone-specific median OS regarding anthracycline sensitivity. OS. OS, overall survival.

Pixantrone was applied as the third line treatment in 3 patients (25%), the forth line treatment in 6 patients (50%), the fifth line treatment in 1 patient (8%) and the eighth line treatment in 2 patients (17%) (Table I). Eight patients received multidrug treatment with PREBEN, 2 patients with PEBEN (CD20-negative relapse of lymphoma) and 2 patients were treated with pixantrone monotherapy. The median number of cycles was 2, range, 1–6. Two patients had minor response and 1 patient stable disease at mid-treatment evaluation, but progressed during further treatment. Other 9 patients experienced disease progression already during the first (3 patients) or the second cycle (6 patients). Four patients received palliative radiotherapy for progression following pixantrone containing treatment. None of the patients was alive at the time of analysis and all patients died of progressive lymphoma. The median OS for the whole group was 21 months (range, 8.5–230 months) (Fig. 1A) while pixantrone specific median OS was 3.5 months (range, 0.5–10 months) (Fig. 1B). A somewhat superior median OS (P=0.065) and median pixantrone specific OS (P=0.593) were observed in patients primarily sensitive to anthracyclines (Fig. 2A and B) yet the statistical significance/insignificance could be considered unreliable due to a limited number of patients established as anthracycline sensitive.

#### Toxicity data

All patients underwent cardiac investigation with cardiac ultrasound or radionuclide ventriculography prior to introduction of pixantrone therapy. There were no episodes of cardiac toxicity observed during the pixantrone treatment. One patient experienced arrhythmia after the last pixantrone infusion, which was attributed to electrolyte imbalance (hypophosphatemia and hypomagnesemia).

Despite the applications of granulocyte colony-stimulating factors in all patients, we observed 4 episodes of grade IV neutropenia and 1 episode of febrile neutropenia. Two patients without significant neutropenia presented with bacterial pneumonia and one with urosepsis. As to the hematological toxicity, we observed 6 episodes of thrombocytopenia grade III and IV in 4 patients and grade III anemia in 1 patient, requiring supplementation of blood products. Chemotherapy had to be delayed for 1 week in only 3 patients, and in 1 patient for 2 weeks, due to unresolved hematological toxicity.

## Discussion

Our results present another perspective of pixantrone treatment and confirm the observations of the retrospective UK study (13). In PIX301 study pixantrone was effective in terms of OS when applied as the third- or fourth line treatment of relapsed or refractory DLBCL patients, but not when used as the fifth line or later (11). Compared to the results of this pivotal study, where an excellent outcome for the advanced line treatment has been observed (11), our real world results as well as the results of the UK retrospective analysis (13) were not so satisfactory. The discordance of our and the UK analysis results (13) with the PIX301 results (11) (Table II) suggests the importance of correct selection of patients for treatment with pixantrone. Firstly, we need to stress that the histological type of lymphoma in our patients as well as the high disease stage at diagnosis and high IPI scores in majority of the patients definitively represented negative predictors of the disease course. Moreover, the high proportion of primarily refractory disease (58%) reflecting also the refractoriness to anthracyclines, additionally predicted a dismal course. Further, in our study only 3 patients were treated with pixantrone in the third line treatment while others received the treatment in later lines of therapy. Interestingly, the patients' characteristics in the UK analysis (13) were much more comparable, actually reflecting the real world setting.

It is unquestionably noteworthy that in the PIX301 study, the inclusion criteria was a complete or relative response to anthracycline treatment lasting at least 24 weeks (11). Our group of patients included as much as 75% of patients who were insensitive to anthracycline treatment by the definition of the PIX301 study. Confirming, those of our patients who responded adequately to first line anthracycline treatment (25%) had a somewhat longer OS compared to those who did not (Fig. 2A; P=0.065). In spite of this, we observed no difference in pixantrone specific median OS while comparing the anthracycline sensitive and insensitive groups (P=0.593). In the UK observation study as much as 71% of patients were sensitive to prior anthracycline treatment while only 29% were not (13). Accordingly, their pixantrone specific OS regarding response to anthracycline treatment was significantly different between the sensitive and insensitive group (P=0.01). The anthracycline response of >24 weeks was also the univariate predictor of progression or death in their analysis, yet not confirmed in the multivariate analysis (13).

On the other hand, a recent extended survival analysis of the PIX301 trial showed that the outcome of treatment with pixantrone is not related to prior treatment outcome (12). Still, despite the results of this analysis (12), we believe that refractoriness to anthracyclines as the predictor of response to pixantrone merits further investigation.

When comparing the median OS rates, it was correspondingly longer in the PIX301 study-10.2 months (11) paralleled to only 3.5 months in our real world study. However, it was even shorter in the UK retrospective analysis (13). The main reason may lie in the inappropriate patient selection for pixantrone treatment in the real world setting, as already stated above-with high risk histological types, high disease stages, having high proportion of primary refractory disease and receiving late lines of treatment with pixantrone. According to the three multivariate predictors of longer progression free survival in the UK analysis (13), our patients belonged to the prognostically worst group-including no patients with relapsed disease but solely those with refractory one, including 58% of primarily refractory patients and including 75% of those who received three or more previous chemotherapies.

The distribution of histological subtypes in our group of patients was somewhat against favor of the ABC subtype compared to the data from the literature (15). This subtype has been otherwise associated with a less favorable outcome when treated with R-CHOP therapy as the frontline treatment (16). However, the universal BCL2 expression in all samples seems to be alarming, predicting a higher probability of resistance to systemic treatment and a lesser OS (17–19). Furthermore, the prevalent high proliferative activity in our series most probably also contributed to the poorer clinical outcomes (20–22). Certain patients with more indolent and lower risk disease might do relatively well with other palliative therapies, but we need to underline that clinical characteristics of our patients as well as histopathological features of their DLBCLs were far from indolent or low risk.

Therefore, to improve the survival outcomes, it would be most beneficial if patients, predicted to have a worse outcome (CD20-negative, BCL2-positive, with high proliferative activity), were treated differently upfront-potentially with an inclusion of a BCL2 inhibitor combined to chemotherapy.

Most common adverse effects of pixantrone include cardiac toxicity and bone marrow suppression, particularly of the neutrophil lineage. The incidence of severe infections, however, is usually low and other toxicities, such as nausea, vomiting, and diarrhea, are rather infrequent and usually mild (11). Both the PIX301 study (11) and the UK analysis (13) reported the occurrence of adverse events consistently with what was expected in heavily pretreated patients receiving a cytotoxic agent. Our observations were also quite similar.

In conclusion, relapsed or refractory DLBCL is an aggressive disease where long term remissions are hard to achieve and every line of treatment brings shorter responses. Novel agents like pixantrone have been shown to improve disease-free and OS, but only in a highly selected population of patients. According to our real life outcomes an appropriate selection of patients for this treatment is crucial and we recommend considering patients with relapsed (and not refractory) disease, patients with non-primary refractory disease and those with fewer lines of prior therapy. Bearing in mind also the still existent (yet reduced) risk of cardiotoxicity, as well as hematological toxicity of pixantrone, we would advise against using it to treat patients with primary anthracycline response lasting <24 weeks and those who were refractory to last treatment. Nevertheless, our conclusions need to be considered with caution due to our limited patient sample. Still, histology needs to be considered when deciding about the first (and future line) treatments.

## Figures and Tables

**Table I.** Patient demographical data and histopathological characteristics of patient samples.

Sex and age (years)	First therapy	Number of previous lines of therapy	Histological type of lymphoma	ABC or GCB subtype	CD20 expression^a^	BCL2 expression^b^	Proliferative activity (%)^c^	Double expressor DLBCL	*C-myc* rearrangement
F, 65	R-CHOP + MD MTX, then R-EPOCH	2	Asian variant of intravas cular large B-cell lymphoma	ABC	+	+	95	−	−
F, 36	CHOP	7	Low grade follicular lymphoma, transformed into DLBCL NOS	GCB	+	+	40	−	−
M, 63	R-CHOP + MD MTX	3	DLBCL NOS	GCB	±	+	>95	−	+
F, 55	R-CHOP	3	DLBCL NOS	GCB	+	+	90	−	+
F, 76	R-CHOP	3	DLBCL NOS	ABC	+	+	70	−	−
M, 77	R-CHOP	3	DLBCL NOS	GCB	+	+	80	−	−
F, 65	R-CHOEP	2	DLBCL NOS	ABC	+	+	>90	−	−
F, 66	R-CHOP	7	DLBCL NOS	ABC	+	+	>80	−	−
F, 73	R-CHOP	2	DLBCL NOS	GCB	+	+	>70	−	−
F, 66	R-CHOP	3	DLBCL NOS	GCB	+	+	>70	−	−
M, 64	R-CHOP	4	DLBCL NOS	ABC	+	+	100	+	−
F, 60	R-CHOP	3	DLBCL NOS	GCB	+	+	90	−	−

a ± simultaneously one lymph node sample was CD20-negative, while the other lymph node sample was positive disproportionately in <50% of cells

b + BCL-2 expressed in >50% of cells

c Proliferative activity is expressed as % of Ki67^+^ cells. F, female; M, male; DLBCL, diffuse large B-cell lymphoma; ABC, activated B-cell; GCB, germinal center B-cell; R-CHOP, rituximab, cyclophosphamide, doxorubicin, vincristine, prednisolone; MD MTX, middle dose methotrexate; R-EPOCH, rituximab, etoposide, prednisolone, vincristine, cyclophosphamide, doxorubicin; CHOP, cyclophosphamide, doxorubicin, vincristine, prednisolone; R-CHOEP, rituximab, cyclophosphamide, doxorubicin, vincristine, etoposide, prednisolone; NOS, not otherwise specified.

**Table II.** Patient characteristics and outcomes, a comparison between the PIX301 study, UK retrospective analysis and the present study.

Variable	PIX301 study	UK analysis	Present study
Number	70	90	12
Median age, years	60	66	65
Males, %	66	66	25
Stage III/IV, %	73	90	100
IPI score ≥2 immediately prior to pixantrone treatment, %	70	94	100
≥3 ChT prior to pixantrone treatment, %	54	34	75
Sensitive to previous anthracyclines^a^, %	100	71	25
Previous treatment with rituximab, %	54	99	100
Duration of first response <12 months, %	0	40	92
Refractory to last treatment^a^, %	57	85	100
Overall response rate	CR 20%,	CR/Cru 10%,	ORR 0%
	PR 17%=ORR 37%	PR 14%=ORR 24%	
Median progression-free survival, months	5.3	2.0	NA
Median pixantrone-specific overall survival, months	10.2	3.4	3.5

a According to criteria of the PIX301 study. IPI, international prognostic index; ChT, chemotherapy regimens; CR, complete response; Cru, complete response unconfirmed; PR, partial response; ORR, overall response rate.

## Data Availability

The datasets used and/or analyzed during the current study are available from the corresponding author on reasonable request.
